# Development of fast patient position verification software using 2D-3D image registration and its clinical experience

**DOI:** 10.1093/jrr/rrv032

**Published:** 2015-06-16

**Authors:** Shinichiro Mori, Motoki Kumagai, Kentaro Miki, Riki Fukuhara, Hideaki Haneishi

**Affiliations:** 1 Research Center for Charged Particle Therapy, National Institute of Radiological Sciences, Inage-ku, Chiba 263–8555, Japan; 2 Research Center for Frontier Medical Engineering, Chiba University, Japan

**Keywords:** radiotherapy, patient set-up, 2D/3D registration, GPU, interfractional changes

## Abstract

To improve treatment workflow, we developed a graphic processing unit (GPU)-based patient positional verification software application and integrated it into carbon-ion scanning beam treatment. Here, we evaluated the basic performance of the software. The algorithm provides 2D/3D registration matching using CT and orthogonal X-ray flat panel detector (FPD) images. The participants were 53 patients with tumors of the head and neck, prostate or lung receiving carbon-ion beam treatment. 2D/3D-ITchi-Gime (ITG) calculation accuracy was evaluated in terms of computation time and registration accuracy. Registration calculation was determined using the similarity measurement metrics gradient difference (GD), normalized mutual information (NMI), zero-mean normalized cross-correlation (ZNCC), and their combination. Registration accuracy was dependent on the particular metric used. Representative examples were determined to have target registration error (TRE) = 0.45 ± 0.23 mm and angular error (AE) = 0.35 ± 0.18° with ZNCC + GD for a head and neck tumor; TRE = 0.12 ± 0.07 mm and AE = 0.16 ± 0.07° with ZNCC for a pelvic tumor; and TRE = 1.19 ± 0.78 mm and AE = 0.83 ± 0.61° with ZNCC for lung tumor. Calculation time was less than 7.26 s.The new registration software has been successfully installed and implemented in our treatment process. We expect that it will improve both treatment workflow and treatment accuracy.

## INTRODUCTION

Conventional patient set-up is commonly performed using a tattoo on the patient's skin to ensure correct set-up using a laser localizer. Recently, several treatment centers have started using a laser marker on the treatment room wall/ceiling or X-ray imaging system [1]. Recent improvements in high conformal irradiation treatment techniques require high positioning accuracy, but patient positioning takes several minutes to complete. In particular, adjustment of the rotational component of the coordinate transformation (yaw, pitch, roll) is more difficult than that for coordinate transformation (left–right, anterior–posterior and superior–inferior). In our existing treatment building (HIMAC), patient position is verified by landmark-based manual registration using orthogonal flat panel detector (FPD) X-ray systems, but this takes up to several tens of minutes to complete [2]. A recently introduced patient positional system uses the 2D-3D image registration technique with a combination of 2D images and the volumetric CT data used for treatment planning, and calculates the registration error between the treatment and planning stages. Although commercial systems for photon beam therapy such as Cyberknife and ExacTrac already provide a 2D-3D auto-registration function for patient set-up [3], integration and customization of the registration function in particle therapy is particularly difficult, because most of them supported digital reconstructed radiography (DRR) as a reference but not X-ray images (discussed in later).

Our treatment center has completed a new treatment facility for carbon-ion beam scanning treatment, and began clinical trials in mid-May, 2011. This facility was designed to improve several aspects of the treatment process. For one of these, we developed a new software application for patient positional verification (called ‘ITchi-Gime (ITG)’ in Japanese) with auto/manual registration. Here, we evaluated the basic performance of this system using clinical data obtained in our new facility.

## MATERIALS AND METHODS

### Carbon-ion beam treatment workflow

Treatment workflow for carbon-ion beam treatment is similar to that for proton beam and external beam photon therapy. The design of our new treatment facility was focused on providing a treatment workflow that could be adapted for use in any treatment situation. The main workflow consists of immobilization, CT acquisition, treatment planning, simulation, quality assurance, and treatment beam irradiation.

**Fig. 1.**
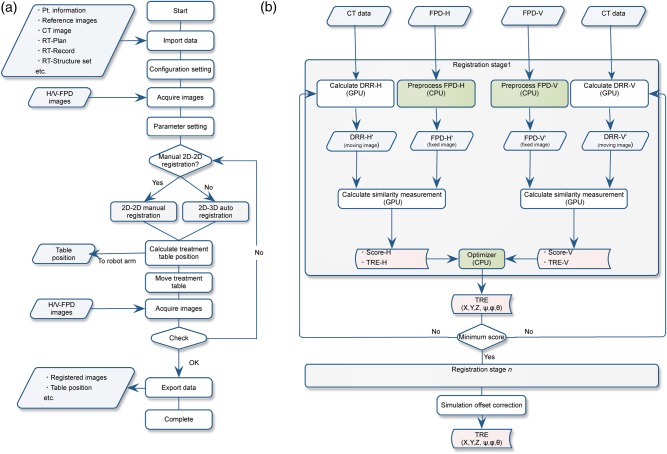
(**a**) Patient positional verification flow chart incorporating the 2D/2D-ITG and 2D/3D-ITG functions. (**b**) 2D/3D-ITG function computation flow chart. White and green boxes show GPU and CPU calculations, respectively. Abbreviations: TRE = target registration error, CPU = central processing unit, GPU = graphics processing unit, FPD = flat panel detector.

First, the patient enters the preparation room to change into an examination gown and undergo any necessary preprocessing. The patient then lies on the treatment table on the shuttle for transfer to the simulation/treatment room, or walks there him- or herself. The robotic arm bed is then transferred to the room isocenter to prepare for patient positional verification and treatment. The system controls the treatment couch with an absolute accuracy of <0.5-mm diameter. Generally, most treatment centers verify patient position in the treatment stage using a virtual simulation process that provides DRR images (mostly beam eye's view) (Fig. 1a).

**Fig. 2.**
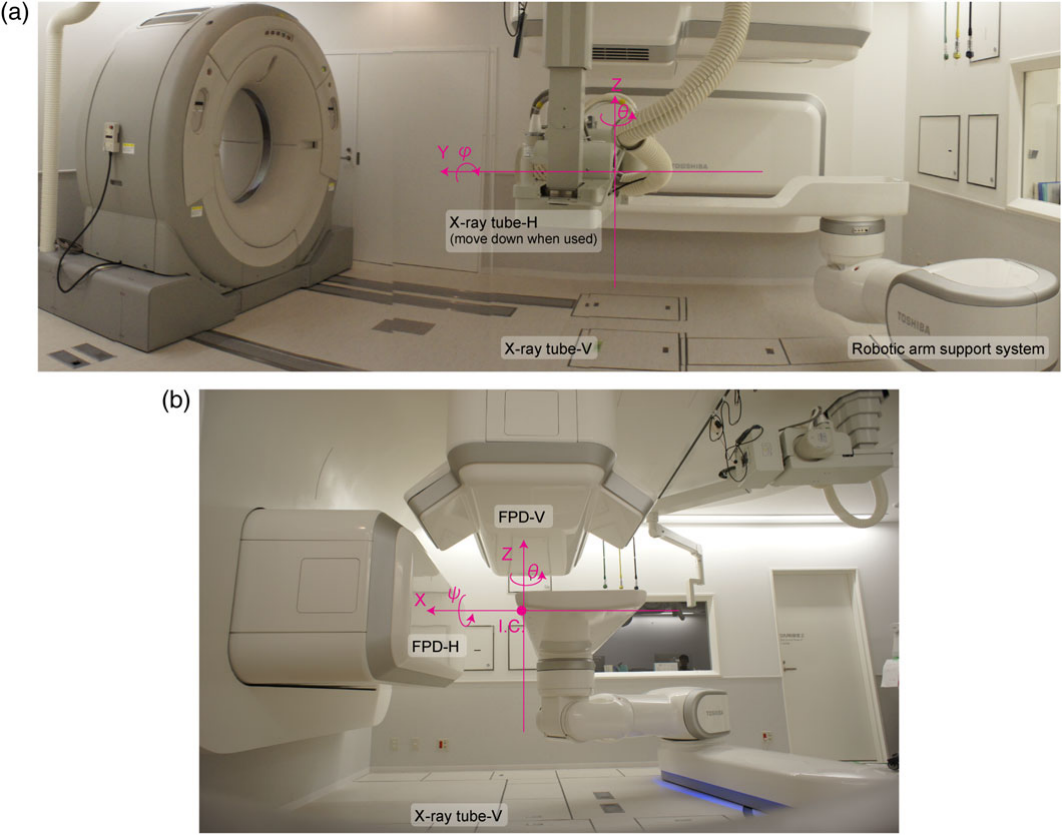
(**a**) Side and (**b**) front views of the simulation room. X-ray imaging system and CT installation positions. Fixed reference coordinates are displayed as pink lines. The distance from the room isocenter (ISO) and source–image receptor distance (SID) are 155 cm and 213 cm, respectively. Abbreviations: I.C. = iso-center position.

In contrast, our center and several other particle centers use a physical simulation process (Fig. 1b). The patient lies on the treatment couch and is transferred to the room isocenter so that the target isocenter position can be matched as defined in treatment planning. The patient positional verification process is done by registering the orthogonal X-ray images to the reference DRR images. The registered X-ray images are used as the reference images during the course of treatment. When non-coplanar irradiation is delivered from the superior aspect of the head (*θ* = 270° in the IEC table-top coordinate system [4]), a horizontal X-ray image cannot be acquired because the horizontal X-ray tube is at risk of collision with the treatment bed (Fig. 2).

Our patient verification process is done in the coplanar position (*θ* = 0 or 180°), even though the 2D-3D auto-registration function requires non-coplanar irradiation for the acquisition of orthogonal X-ray images. The ITG sends the next couch position to the robotic arm support system, and orthogonal X-ray images are again acquired to verify patient position for simulation offset correction (SOC). If necessary, registration is done manually by the therapist. This results in a discrepancy between the actual treatment table position and that calculated by the auto-registration function, which allows final registered orthogonal FPD images to be obtained. After the verification of patient position, X-ray images are preferably acquired as a record. After irradiation with the treatment beam, the patient exits the treatment room for the preparation room and changes out of the examination gown. All treatment workflows are managed by the treatment management system (TMS) via the sending and receiving of order messages and responses.

### Registration algorithm

The ITG includes two functions, 2D/2D manual registration (2D/2D-ITG) and 2D/3D auto-registration (2D/3D-ITG). These two functions work interactively. Here, we focus on 2D/3D-ITG.

**Fig. A1.**
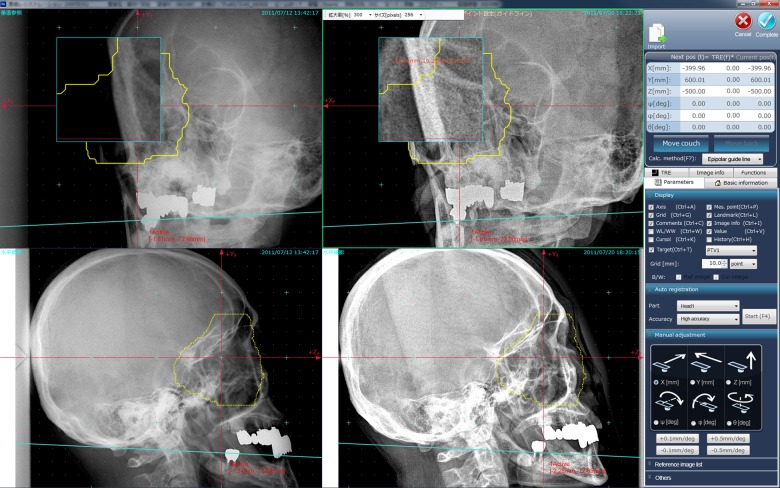
Main screenshot of the ITG software. Left and middle panels show reference and FPD images, respectively. Light blue lines are epipolar lines for support in selecting the same specific point in 3D space. Yellow line shows the planning target volume (PTV).

For the 2D-2D registration function, separate manual registration using the point match strategy does not provide correct registration because the respective image plane involves three parameters (two translations (Xr, Yr) and one rotation (θr) in plane). We therefore integrate epipolar geometry to obtain a specific position in 3D space (light blue lines in Fig. A1).

2D/3D-ITG is performed using two FPD images from different directions—any two different directions are possible, but the vertical and horizontal directions were selected for this study—and the planning 3DCT dataset [5]. This algorithm registers DRR images projected by the 3DCT data to the acquired horizontal/vertical FPD images and derives 6DOF positional error values. The computation flow chart is summarized in Fig. 1b. 2D/3D-ITG uses GPU (graphic processing unit) computation as a parallel computational architecture for accelerating computation time while maintaining calculation accuracy [6]. The ITG software is programmed using CUDA (Compute Unified Device Architecture) ver. 4.0 on Microsoft Visual Studio 2010 (Microsoft Corp., Redmond WA, USA) in a Windows 7 environment with the GPU processor on an NVIDIA TESLA C2075 board (NVIDIA Corporation, CA, USA). This board is equipped with 448 CUDA core units, a 1.15 GHz core clock, a 1.5 GHz memory clock, and 6 GB of memory, and provides a processing speed of more than 1.03 Tflops for single precision calculation. To improve calculation time, we used the Intel integrated performance library (IPP) and Intel math kernel library (Intel Corporation, Santa Clara, CA). The ITG software is installed on a workstation (Dell, Precision R5400, 2.66 GHz dual quad-core CPU Intel processor, 16 GB physical memory).

#### Preprocessing of FPD images

Imported FPD images (FPD-H and FPD-V) are resized to the same pixel size as the DRR images. Image processing is applied to emphasize the edge of the structure and reduce interpolation artifacts [7]. The output of this process is the resulting FPD images (FPD-H' and FPD-V'), and the process is performed for FPD images in every acquisition.

#### Projecting DRR images

DRR calculation is the summation of CT voxel values along the X-ray projection ray. Although several DRR calculation algorithms have been reported [8, 9], our implementation [10] was designed to improve calculation speed based on the extension of a Siddon ray tracing algorithm [9] to volume data using trilinear interpolation to reduce interpolation errors [7]. To provide similar image quality to FPD, we convert these CT voxel data to the X-ray attenuation for photon energy using image processing of the CT-number weighting in each CT voxel data before integration of the pixel value along the ray. Another fast calculation technique segments the CT data within the patient surface and integrates CT voxel values within this region only. Moreover, we set calculation ROIs on orthogonal FPD images, which are user-selected registration regions, on the basis that patient anatomical position may not be exactly the same in respective treatment fractions [10]. If the user forgets to set the calculation ROI, the ITG automatically sets it by expanding the target ROI regions. DRR calculation is done within the above regions only. These calculation regions are recorded in an RT image, and automatically displayed during the treatment course. These two techniques each provide a significant reduction in computation cost.

#### Parameter optimization

Parameters described here are DRR projection parameters (six degrees of freedom (6DOF)), which iteratively estimate the unknown pose of the X-ray system relative to the CT volume. These parameters are defined using normalized mutual information (NMI) [11], gradient differences (GDs) [5], and zero-mean normalized cross-correlation (ZNCC) as similarity measures (see Appendix). We used a single or combination of these metrics to calculate the final score by applying weighting values to the respective scores for each anatomical site. 2D/3D-ITG calculates the four respective parameters from each image direction, namely the registration errors for *X*, *Y*, *φ* and *θ* and those for *Y*, *Z* and *ψ*, as derived from the vertical and horizontal images, respectively. This is because positional errors in translation and rotation on the plane are more easily recognized than those out of the plane (Fig. 1). For example, positional errors in *Y*, *Z* and *ψ* are easy to recognize in horizontal images. Therefore, weaker directions (e.g. *X* and *θ* in horizontal images) in respective images are supported by each other. Since vertical and horizontal images share a Y-axis, registration errors in *Y* and *φ* derived from respective images are averaged. This averaging allows the calculation of 6DOF registration errors. However, when two FPDs are installed in any configuration apart from 90°, 2D/3D-ITG calculates the six parameters from each image direction.

To minimize the number of iteration calculations, 2D/3D-ITG uses a conjugate gradient descent optimizer (Powell–Brent method [12] and L-BFGS method), which minimizes the cost function by calculating the exact line search optimization and outputting the next DRR projection parameters. It then recalculates the DRR images, excepting when the DRR projection parameter is a small translation or rotation in the plane, in which case the DRR image is not recalculated but rather the original image is translated or rotated. This iterative process is repeated for a solution by evaluating the cost function at respective positions.

2D/3D-ITG uses a multi-resolution strategy (coarse-to-fine) to improve computational speed, accuracy and robustness. In this study, we applied two stages (down sampling of the original CT by a factor of (x,y,z) = (3,3,3), (2,2,2) and (1,1,1)). DRR calculation grid space also changes in respective stages. For the NMI calculations, 64 histogram bins were used for the images at all resolution stages. As described in the previous section, calculation accuracy in ZNCC was improved when DRR image quality was close to that of FPD.

If the therapist or oncologist finds the registration result unsatisfactory, the user can perform positional fine-tuning manually by observation after completing the 2D/3D-ITG function to match the acquired X-ray images (2D-3D manual registration) to minimize patient positional error to avoid degrading carbon-ion dose conformation. Since this process is performed as a real-time operation, it is useful in helping users understand registration errors.

### Data acquisition

Orthogonal FPD images are acquired in two sets using an X-ray imaging system (Canon CXDI-55C, Tokyo, Japan) with an imaging area size of 35 cm × 43 cm and a pixel pitch of 0.16 mm. A CsI scintillator receptor is used for static image acquisition. All FPDs are installed within the port cover. The vertical X-ray tube is set under the floor (Fig. 2), and the horizontal X-ray tube is set at the opposite side of the horizontal FPD and moved down when it is used. The distance from the room isocenter (ISO) and source–image receptor distance (SID) are 155 cm and 213 cm, respectively.

Regarding CT scan conditions for patients, CT imaging is performed in helical mode using a 16-multi-slice CT scanner (MSCT) on rails (Aquilion LB, Toshiba Medical Systems, Otawara, Japan) under free-breathing conditions for CT of the head, and with breath-holding at exhalation for CT of the pelvis. Scan conditions are based on clinical conditions and a pitch factor of 15 and slice collimation of 16 × 2.0 mm. For reconstruction parameters, the pixel size is 1.074 mm. For lung and liver cases, the volumetric cine CT is acquired with a 320MSCT (Aquilion One Vision, Toshiba Medical System, Otawara, Japan). CT scan conditions were a tube voltage of 120 kV, 70–100 mA, 0.5 s per rotation, and slice collimation of 280 × 0.5 mm. Since the scan range of the 320MSC in a single rotation (∼16 cm) is insufficient to cover the whole lung, a few 4DCT scans were acquired and the couch was moved to the next position. Reconstruction parameters were a pixel size of 0.977 mm and a slice thickness of 1.0 mm.

### Evaluation methods

We evaluated the 2D/3D-ITG function in terms of computation time and registration accuracy. Registration errors were defined using the similarity metrics GD, NMI, ZNCC and their combination. These metrics have various characteristics, as described in the Appendix and can be strongly affected on the image quality (bone emphasizes sites such as pelvic and head regions and soft tissue emphasizes sites such as thoracic and abdominal regions). Therefore, we combined these metrics to complement respective similarity metrics characteristics. The study enrolled 21 patients with head and neck tumors, 20 with prostate tumors and 12 with lung tumors receiving carbon-ion beam treatment who were randomly selected from among patients at the NIRS. Performance of the 2D/3D-ITG function was evaluated using the planning CT data and final registered orthogonal FPD images in actual treatment. SOC values were all zero in this study because we used DRR images as reference images. Since the positional accuracy of the treatment couch is <0.5 mm, but not zero, movement of patient position by the treatment couch might have resulted in residual positional error, even after completion of the patient set-up procedure. Positional error between the CT data and the final registered FPD images was calculated using the 2D/3D-ITG function, and the resulting values were defined as the registration offset value. The CT data were then shifted (*x*, *y*, *z*, *ψ*, *φ*, *θ*) = (20 mm, 20 mm, 20 mm, 2°, 2°, 2°) along the room axis coordinate defined by IEC61217. The 2D/3D-ITG function was started, and the registration offset value was subtracted from the resulting values, with TRE and angular error (AE) expressed as follows:

$$\text{TRE}=\sqrt{\Delta x^{2}+\Delta y^{2}+\Delta z^{2}}\;,\;\;\;\;\;\;\;\;\;\mathrm{AE}=\sqrt{\Delta \psi^{2}+\Delta \varphi^{2}+\Delta \theta^{2}}.$$

Since registration error using six parameters (three translations and three rotations) was not expressed directly, we used TRE and AE, although with the disadvantage that expressing them this way made them appear slightly larger than the parameters themselves. In any case, the three translational errors should be < 0.58 mm to achieve a TRE value of <1.0 mm.

## RESULTS

### Head and neck tumor patient

**Fig. 3.**
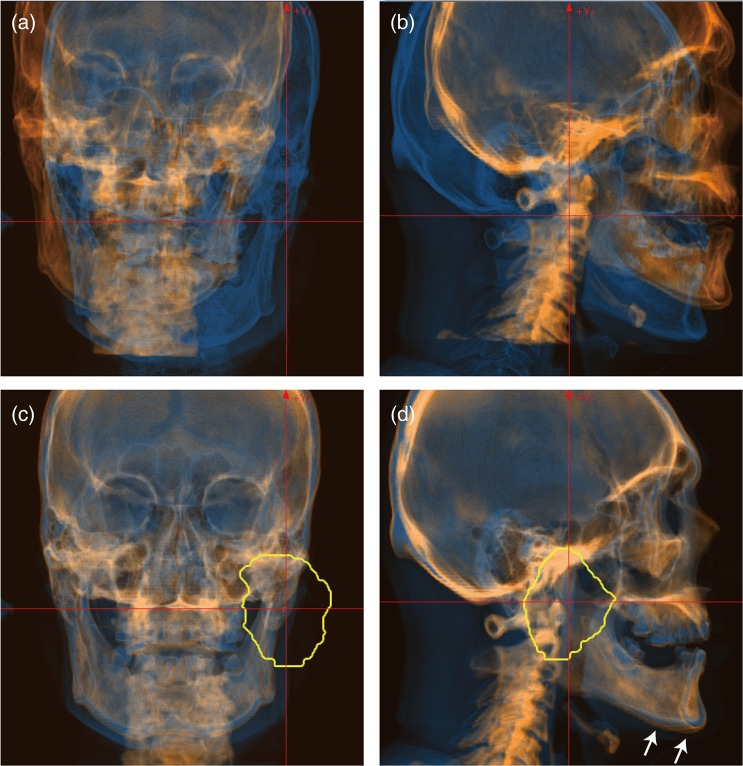
FPD images (blue layer) overlaid on DRR images (orange layer). (**a**) Vertical and (**b**) horizontal images before registration, and (**c**) vertical and (**d**) horizontal images after registration for a patient with a head and neck tumor. Registration metric was used ZNCC + GD. Yellow line shows planning target volume (PTV). White arrows in the lower panels are positional errors, which differ from the referenced DRR images.

Figure 3 shows a screen shot of the automatic registration function for a patient with a head and neck tumor. Reference DRR and acquired X-ray images are colored orange and blue, respectively. The registration metric with GD produced the worst registration results (TRE = 25.89 mm and AE = 2.23°). The registration accuracy with NMI (TRE = 0.91 mm and AE = 0.44°) was not improved by adding the GD metric (TRE = 1.04 mm and AE = 0.64°). The best registration accuracy was TRE = 0.60 mm, AE = 0.26°, with the registration metric of ZNCC + GD. Further, this was improved by adding the GD metric to NMI (TRE = 1.54 mm and AE = 0.93°). Since the planning target volume (PTV) was located around the right jaw joint, we set the calculation regions of interest (ROIs) around this location. The registered images produced by the auto-registration process, displayed in the lower panels of Fig. 3, show that the reference and acquired images are well registered around the PTV. Since the upper/lower jaw and neck positions in patient set-up before irradiation differ from those in the acquired planning CT, these positions were not well registered (marked with white arrows in Fig. 3d).

For all head and neck patients, the registration metrics parameter dependencies were relatively small, except those of GD (Table 1). The average registration errors were TRE = 35.43 ± 4.46 mm and AE = 4.18 ± 1.37° for the GD metrics. The registration accuracy with the NMI metric was TRE = 0.81 ± 1.07 mm and AE = 0.58 ± 0.63°, but was further degraded to TRE = 3.05 ± 8.13 mm and AE = 1.63 ± 4.23° on combination of GD. The registration error for the ZNCC + GD metrics was the smallest of all (TRE = 0.45 ± 0.23 mm and AE = 0.35 ± 0.18°), and metrics that included ZNCC showed improved registration error, whereas those that included NMI did not. Computation time was increased by combination with GD (e.g. 5.78 ± 0.96 s with ZNCC, 7.26 ± 1.41 s with ZNCC + GD). Computation time with the GD metric was shortest of all (0.87 ± 0.22 s), because registration was finished due to the local minimum in the optimization process.

**Table 1.** Summary of registration accuracy

			TRE (mm)	AE (deg)	Time (s)
No.	Anatomical site	Metrics	Mean ± SD	Mean ± SD	Mean ± SD
1	Head & neck	GD	35.43 ± 4.46	4.18 ± 1.37	0.87 ± 0.22
2		NMI	0.81 ± 1.07	0.58 ± 0.63	5.07 ± 1.88
3		NMI + GD	3.05 ± 8.13	1.63 ± 4.23	6.93 ± 1.46
4		ZNCC	0.52 ± 0.35	0.40 ± 0.26	5.78 ± 0.96
5		ZNCC + GD	0.45 ± 0.23	0.35 ± 0.18	7.26 ± 1.41
6	Pelvis	GD	37.44 ± 6.74	4.04 ± 1.69	0.75 ± 0.17
7		NMI	0.27 ± 0.21	0.19 ± 0.12	3.04 ± 0.92
8		NMI + GD	0.21 ± 0.10	0.17 ± 0.10	4.83 ± 1.18
9		ZNCC	0.12 ± 0.07	0.16 ± 0.07	3.75 ± 1.04
10		ZNCC + GD	0.15 ± 0.12	0.14 ± 0.05	4.90 ± 1.18
11	Lung	GD	31.69 ± 16.27	3.91 ± 2.25	0.72 ± 0.24
12		NMI	3.58 ± 6.50	2.30 ± 4.08	4.61 ± 2.09
13		NMI + GD	1.56 ± 1.45	0.92 ± 0.71	6.54 ± 2.38
14		ZNCC	1.19 ± 0.78	0.83 ± 0.61	4.83 ± 1.97
15		ZNCC + GD	1.45 ± 1.48	0.94 ± 0.87	5.59 ± 2.33

TRE = target registration error, AE = angular error, SD = standard deviation, GD = gradient difference, NMI = normalized mutual information, ZNCC = zero-mean normalized cross-correlation.

### Pelvic tumor patient

**Fig. 4.**
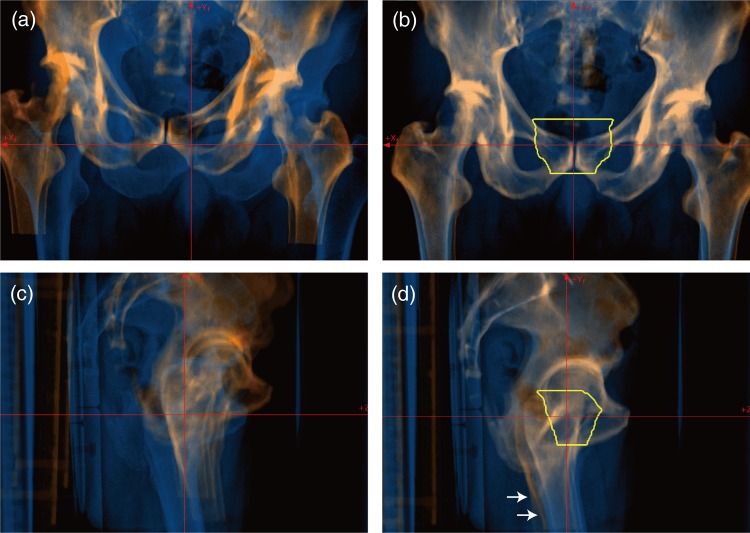
FPD images (blue layer) overlaid on DRR images (orange layer). (**a**) Vertical and (**b**) horizontal images before registration and (**c**) vertical and (**d**) horizontal images after registration for a patient with a pelvic tumor. Yellow line shows planning target volume (PTV). Registration accuracy was TRE = 0.1 mm and AE = 0.1° with ZNCC + GD. White arrows marked in lower panels are positional errors, which differ from the referenced DRR images.

Orthogonal FPD images obtained before and after registration are shown in Fig. 4. FPD and referenced DRR images show good registration, but the positions of the femoral bones on both sides were not registered (white arrows in Fig. 4d) because the reproducibility of femoral bone positional was low. When the femoral bones were not correctly registered, the medical staff registered the femoral bone on the treatment direction side. The GD metric caused a large calculation error (TRE = 31.42 mm and AE = 3.72°), as was also seen in the head and neck tumor patient, whereas registration accuracy with the other metrics was almost the same (TRE < 0.24 mm, AE < 0.22°, calculation time < 5.34 s).

For all pelvic tumor patients, registration accuracy was independent of all metrics except GD, and was less than TRE = 0.27 ± 0.21 mm and AE = 0.19 ± 0.12°. Computation time was less than 4.90 ± 1.18 s (Table 1).

### Lung tumor patient

**Fig. 5.**
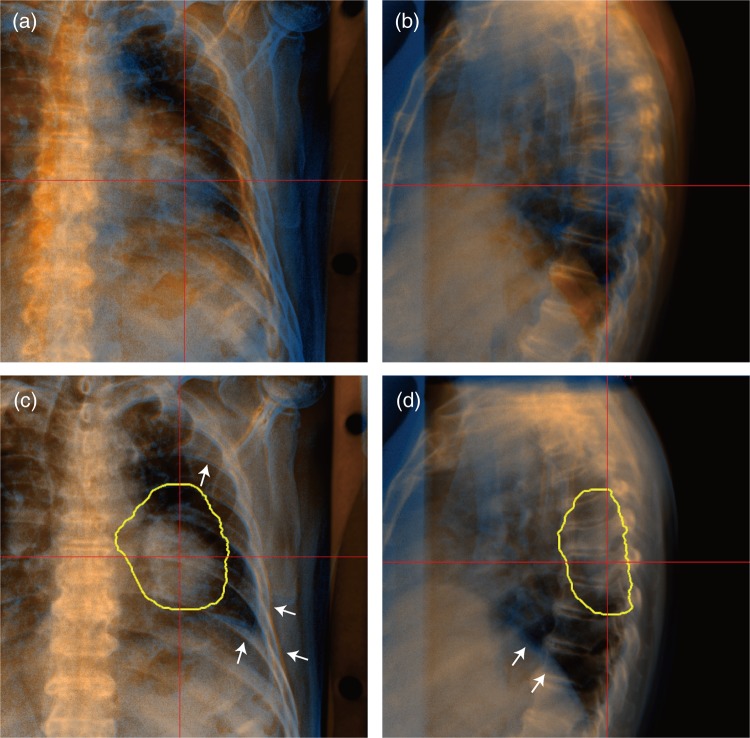
FPD images (blue layer) overlaid on DRR images (orange layer). (**a**) Vertical and (**b**) horizontal images before registration and (**c**) vertical and (**d**) horizontal images after registration for a patient with a lung tumor. Registration accuracy was TRE = 0.4 mm and AE = 0.3° with NMI + GD. Yellow lines shows planning target volume (PTV). White arrows marked in Fig. 5c and d are rib and diaphragm positional errors due to respiration, which differ from the referenced DRR images.

Although the FPD and DRR images showed large positional differences before registration (Fig. 5a and b), 2D/3D-ITG successfully registered them well (Fig. 5c and 5d). FPD and CT images were acquired at the same respiratory phase (around exhalation), but rib and diaphragm positions were not exactly the same due to limitations in respiratory phase reproducibility (white arrows in Fig. 5c and d). In this case, registration accuracy with ZNCC was almost the same as that with ZNCC + GD (TRE = 2.4 mm and AE = 1.3°). In contrast, registration accuracy was improved using NMI (TRE = 0.65 mm, AE = 0.29° with NMI and TRE = 1.09 mm, AE = 0.51° with NMI + GD). The results with the GD metric were TRE = 32.27 mm, AE = 2.67°.

For all lung patients, registration accuracy with the metric of NMI + GD (TRE = 1.56 ± 1.45 mm and AE = 0.92 ± 0.71°) was better than that with NMI (TRE = 3.85 ± 6.50 mm and AE = 2.30 ± 4.08°) (Table 1). In contrast, accuracy with GD only resulted in large errors, as was also seen in other anatomical sites. Best registration accuracy was TRE = 1.19 ± 0.78 mm, AE = 0.83 ± 0.61° with the metric of ZNCC, with a computation time of 4.83 ± 1.97 s. These results were better than with ZNCC + GD (TRE = 1.45 ± 1.48 mm and AE = 0.94 ± 0.87°), albeit that the differences were small.

## DISCUSSION

We developed a new registration software application with an automatic registration function and integrated this application into a clinical protocol. Registration accuracy was dependent on metric and anatomical site, but was less than TRE = 0.45 mm and AE = 0.35° for the head/neck and pelvic regions and TRE = 1.19 mm and AE = 0.83° for the thoracic region with the optimum registration metrics. Computation time for 2D/3D-ITG was less than ∼7.3 s. These results were found to be acceptable in clinical situations using patient data with intra/interfractional changes.

Positional accuracy with the 2D/3D-ITG algorithm was dependent on calculation ROI. In the head and neck case (Fig. 3), for example, the tumor was located around the jaw joint. Since positional reproducibility for the upper and lower jaws is not high, even when a mouthpiece is used, we generally set the calculation ROI in the upper or lower jaw region when the tumor is actually located in the upper or lower jaw only in treatment. In this case, however, the calculation ROI was set to include both the upper and lower jaw regions, and the resulting position might not have been completely correct. The same situation was seen in the pelvic region with inclusion and exclusion of the femur. Nevertheless, it was easier to set the calculation ROI for the prostate patient than for the head and neck patient because the PTV for prostate treatment is not located on the femur.

Positional accuracy for the thoracic anatomical site was lower than that for the head/neck and pelvic sites. The reasons for this can be explained as follows. First, because organ position can be strongly affected by respiration, we acquired FPD images around full exhalation, but this phase is not identical to that in the reference images. Second, we have less 2D/3D-ITG experience for the thoracic and abdominal sites than for head and neck and pelvic sites because carbon-ion scanning beam treatment for these sites has not yet started, as of 2014. Our previous report showed that patient set-up accuracy was improved by experience with 2D/3D-ITG [10]. Positional accuracy will, therefore, likely improve after the start of thoracic and abdominal treatment.

We are currently preparing to provide respiratory gating scanning irradiation with an amplitude-based gating system using two oblique directional DFPD imaging systems (Fig. 1) [13]. In the present configuration, the system uses horizontal and vertical direction FPD images, which aid understanding of patient anatomical structures. Since human anatomical structure is bilaterally symmetrical, particularly for bony structures, horizontal and vertical orthogonal images provide anatomical information in an inefficient way. Oblique directional images by the DFPD system will likely be more efficient for 2D-3D registration.

In this study, we evaluated our in-house software using patient data, and found that its registration accuracy was dependent on registration metric and patient treatment site. In clinical situations, patient positional accuracy should be considered to be an amalgam of registration accuracy, treatment couch positional accuracy (0.5-mm diameter in our hospital) and other inter-/intrafractional changes. Optimum margins that reflect these variables should be added to the target in treatment planning.

In our hospital, 2D/3D-ITG has substantially accelerated the average patient set-up procedure time, from 15 min without it to 5 min with it. This fast set-up should improve patient comfort and reduce patient positional variation during the set-up procedure. Moreover, the increase in the number of patients who can be treated is excellent news for both patients and hospital administrators. For patients, the very small number of carbon-ion beam centers worldwide means long waiting times or the need to select other treatment methods, such as photon beam therapy, surgical operation etc. For hospital administrators, the very high construction and maintenance costs of particle therapy centers raise health expenditures, and thus require higher patient numbers [14]. On simple calculation, while actual treatment procedure time (excluding patient set-up) is 5 min, overall room occupation time (from entering to leaving the treatment room) without 2D/3D-ITG is 20 min, versus only 10 min with it. The number of patients treated per hour might accordingly be doubled using 2D/3D-ITG, from three to six, aiding the recovery of construction and maintenance costs.

One limitation of this study warrants mention. Generally, patient set-up is performed by registration of bony anatomical structures using kV/MV X-ray beams; therefore, other registration techniques not using X-ray imaging, such as surface registration, are not suitable for particle therapy. As several papers have reported, however, tumor position does not always remain the same, even though bony structures do [15, 16]. Several treatment centers, therefore, perform patient set-up based on tumor position, particularly those using photon beams. This is because the prescribed dose in photon beam therapy is defined by the tumor center of mass. In contrast, the prescribed dose in particle beam therapy is defined by the tumor mass, because particle beams stop at a certain depth position [17]. We did not evaluate the dose distribution; however, it could be affected due to patient set-up error because the particle beam is strongly affected by tissue density variations along respective rays, especially bone and implanted metal etc. This is beyond the scope of this study. Even when the tumor position remains the same as the reference, the water equivalent path length from the patient surface to the distal edge of the target can change due to tumor shrinkage, changes in soft tissue thickness etc. This is a limitation for bony-based registration in particle therapy, particularly in thoracic and abdominal sites, which are subject to respiratory-induced movement. Fortunately, however, because we match bony anatomical structures and tumor position to the reference positions at the patient set-up stage, any problems with reproducibility occur in the treatment stage. One approach to this problem is the use of an amplitude-based gating strategy, for example using an X-ray fluoroscopic imaging system [18]. In the present study, we registered bony anatomical structures to the reference using 2D/3D-ITG, and turned the treatment beam on when the tumor moved into the gating window defined in treatment planning. This allowed the treatment beam to be delivered to the target correctly.

An alternative approach to this limitation was used by Kramer *et al*. in proton imaging. These authors used medical specimens to improve image contrast by specification of the particle beam (Bragg peak) [19]. Beam range information can be obtained by acquiring this image in respective water equivalent depths (range telescope) [20]. This technique might allow beam range–based patient set-up and improve treatment accuracy.

## CONCLUSION

This software application was developed in-house and is now in routine clinical use at our new treatment facility. Because it is generally more difficult to manually adjust the rotation axis than the translation axis, the 2D/3D-ITG ability of this software to compute rotation axis is an important advantage. Initial clinical experience indicates that this application reduces total treatment time while maintaining high positional accuracy.

## Figures and Tables

**Fig. A2.**
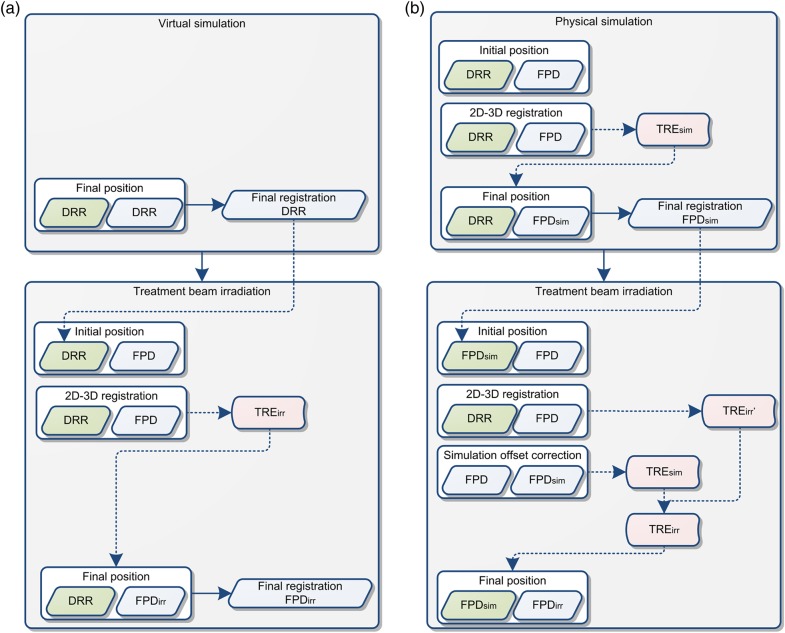
Image types in (**a**) virtual simulation and (**b**) physical simulation workflows. Green, blue and red boxes show reference image, acquired/calculated image and registration result (TRE: target registration error), respectively.

## APPENDIX

### Registration similarity metrics

Registration similarity metrics we used in this study are the NMI [11], GDs [5] and ZNCC as similarity measures, as follows:

$$NMI=\frac{H\left(\mathrm{DRR}\right)+H\left(\mathrm{FPD}\right)}{H\left(\mathrm{DRR},\mathrm{FPD}\right)},$$

where *H*(*DRR*) and *H*(*FPD*) are entropies for DRR and FPD images, and *H*(*DRR*, *FPD*) is the joint entropy. The different modality images are generally registered with NMI, which is more robust than standard mutual information in ensuring the overlapping of very low-intensity regions of the images.

$$GD=\underset{u,v}{\sum }\frac{Const_{v}}{Const_{v}+{\left(\frac{dI_{FPD}}{du}-s\frac{dI_{DRR}}{du}\right)}^{2}}+\underset{u,v}{\sum }\frac{Const_{h}}{Const_{h}+{\left(\frac{dI_{FPD}}{dv}-s\frac{dI_{DRR}}{dv}\right)}^{2}},$$

where *I_DRR_* and *I_FPD_* are DRR and FPD image pixel intensities, respectively, *u* and *v* are orthogonal axes of the image, and *s* is the weighting factor. GD is tentially insensitive to soft tissue deformation but is expected to be sensitive to thin line structures by using intensity gradients.

$$ZNCC=\frac{\Sigma_{u,v}\left(I_{FPD}-\bar{I_{FPD}}\right)\left(I_{DRR}-\bar{I_{DRR}}\right)}{\sqrt{\Sigma_{u,v}{\left(I_{FPD}-\bar{I_{FPD}}\right)}^{2}\cdot \Sigma_{u,v}{\left(I_{DRR}-\bar{I_{DRR}}\right)}^{2}}},$$

where $\bar{I_{FPD}}$ and $\bar{I_{DRR}}$ are pixel intensity mean values for FPD and DRR images, respectively. ZNCC is insensitive to pixel value variation on image background.

### Input/output (I/O) data

The ITG software imports various kinds of format data, including order messages and patient information from the TMS; CT/FPD images, reference images, and DICOM-RT format files from the picture archiving and communication system (PACS); and treatment table position. For the abdominal and thoracic region, after cine FPD images are imported to the ITG software, the user can select the desired respiratory phase. During patient set-up verification, the ITG software exchanges FPD images and table position with the X-ray imaging system and robot arm patient support system (Fig. A1), respectively. On completion of the process, the system then saves orthogonal FPD images to the PACS.

After getting order message from the TMS, the ITG software receives DICOM files from the PACS and sends the initial table position to the robot arm patient support system; absolute table position values described in the RT-Record of physical simulation for cases of physical simulation; and relative position values described in the RT-Plan for cases of virtual simulation.

### Graphical user interface

The ITG software incorporates a graphical user interface, which has four image panels (orthogonal reference and acquired images) and one information panel to facilitate management (Fig. A1). Several visualized functions are integrated; briefly, these include a partial magnifier (which shows a magnification of the original image, not the displayed image, around the cursor), zoom in/out of the whole image area, 4/2 screen modes (to display separate horizontal and vertical images in a larger screen area), image overlay (subtraction, checker board, blend with reference and acquired images in the same direction), etc. Other functions are the projected contour data (derived from the RT-Structure) and measurements (length, angle, pixel value). Generally, the FPD image can be accessed by any image processing application before export to the X-ray imaging system. To ensure image quality in particle therapy, however, we also integrated an in-house image processing function (dynamic range compression (DRC) and multi-frequency processing (MFP)).

Since FPD imaging is in two dimensions, it is difficult to recognize anatomical structures in 3D space. The CT image window is therefore added on the GUI and the 3D point, which the user identifies on the CT image, is projected on the FPD images (Fig. A2). This function is useful in understanding anatomical structures on FPD images, particularly on FPD images acquired in oblique directions.

### Simulation offset correction (SOC)

DRR images are used as a reference in the treatment stage when virtual simulation is done (Fig. A2a). In this case, FPD images are matched to the reference images by moving the treatment table in accordance with the 2D-3D registration results (FPD_irr_ in Fig. A2a).

While physical simulation is done, orthogonal reference FPD images (FPD_sim_ in Fig. A2b) in the treatment stage are acquired in the simulation stage by 2D-3D registration to DRR images (Fig. A2b). Although the 2D-3D registration in the treatment stage calculates the registration results (TRE'_irr_ in Fig. A2b) using DRR and acquired FPD images, this result is not suitable for registration between acquired and reference FPD (FPD_sim_ in Fig. A2b), but rather for registration between acquired FPD and DRR images. In the treatment stage, therefore, registration errors should be corrected by the simulation offset (simulation offset correction: SOC) before the treatment table is moved. In consequence, the treatment table should be moved on both 2D-3D registration results in both the physical simulation and treatment stages (TRE_irr_ = TRE_sim_ + TRE'_irr_ in Fig. A2b).
